# Alteration of Differentiation Potentials by Modulating GATA Transcription Factors in Murine Embryonic Stem Cells

**DOI:** 10.4061/2010/602068

**Published:** 2010-05-11

**Authors:** Callinice D. Capo-chichi, Jennifer L. Smedberg, Malgorzata Rula, Emmanuelle Nicolas, Anthony T. Yeung, Richard F. Adamo, Andrey Frolov, Andrew K. Godwin, Xiang-Xi Xu

**Affiliations:** ^1^Miller School of Medicine, University of Miami, 1550 NW 10th Avenue (M877), Miami, FL 33156, USA; ^2^Department of Medical Science, Fox Chase Cancer Center, Philadelphia, PA 19111, USA; ^3^Department of Surgery, University of Alabama at Birmingham, Birmingham, AL 35294, USA

## Abstract

*Background*. Mouse embryonic stem (ES) cells can be differentiated in vitro by aggregation and/or retinoic acid (RA) treatment. The principal differentiation lineage in vitro is extraembryonic primitive endoderm. Dab2, Laminin, GATA4, GATA5, and GATA6 are expressed in embryonic primitive endoderm and play critical roles in its lineage commitment. *Results*. We found that in the absence of GATA4 or GATA5, RA-induced primitive endoderm differentiation of ES cells was reduced. GATA4 (−/−) ES cells express higher level of GATA5, GATA6, and hepatocyte nuclear factor 4 alpha marker of visceral endoderm lineage. GATA5 (−/−) ES cells express higher level of alpha fetoprotein marker of early liver development. GATA6 (−/−) ES cells express higher level of GATA5 as well as mesoderm and cardiomyocyte markers which are collagen III alpha-1 and tropomyosin1 alpha. Thus, deletion of GATA6 precluded endoderm differentiation but promoted mesoderm lineages. *Conclusions*. GATA4, GATA5, and GATA6 each convey a unique gene expression pattern and influences ES cell differentiation. We showed that ES cells can be directed to avoid differentiating into primitive endoderm and to adopt unique lineages in vitro by modulating GATA factors. The finding offers a potential approach to produce desirable cell types from ES cells, useful for regenerative cell therapy.

## 1. Background

Embryonic stem (ES) cells derived from the inner cell mass of preimplanting embryos can be maintained in vitro and expanded in culture [1, 2]. These pluripotent cells can potentially be differentiated and give rise to every tissue of an organism [3, 4]. The implication for tissue engineering using human ES cells in medical application is tremendous and exploration to manipulate their differentiation into a desirable cell type is being undertaken [4, 5].

ES cells can be induced to differentiate in vitro either by treatment with retinoic acid [6–8] and/or by aggregation [9, 10], and both genetic and extracellular factors influence these processes [11–14]. The main route of differentiation in vitro is the extraembryonic endoderm lineage, which mimics the in vivo differentiation of cells of the inner cell mass to form the primitive endoderm [8, 10, 11]. The GATA transcription factors are expressed in the preimplanting blastocysts and belong to the group of genes that play critical roles in endoderm development [12–19]. GATA4-deleted ES cells are unable to differentiate spontaneously toward the endoderm lineage upon aggregation, but the cells respond to retinoic acid to undergo differentiation [20, 21]. GATA6 is essential for endoderm development and is also required for in vitro endoderm lineage differentiation of ES cells [22, 23]. Transfection/expression of either GATA4 or GATA6 in ES cells is sufficient to induce endoderm differentiation [24, 25], and such analysis led to the suggestion that GATA4 is required for ES cells to sense an aggregation signal, and GATA6 is required to respond to retinoic acid for endoderm differentiation [25]. In contrast to zebrafish and Xenopus in which GATA5 is critical for both endoderm and heart development [26–29], the phenotype of the GATA5 homozygous knockout mice is relatively mild [30]. The differentiation of GATA5-deficient ES cells has not been previously reported.

The primitive endoderm cells are the first epithelial cell type of the embryo that express laminin and collagen IV and produce a basement membrane [31, 32]. Thus, induction of laminin and collagen IV is an indication of endoderm differentiation of ES cells [25]. Oct-3/4 (also known as POU 5F1) is a transcription factor associated with pluripotency of embryonic stem cells, and its expression is lost upon ES cell differentiation [33]. Accompanying ES cell differentiation is the induction of GATA factor expression, which is indicative of ES cell primitive endoderm differentiation [25]. One of the GATA-regulated genes, disabled-2 (Dab2), is an informative marker for the differentiation of ES cells towards the endoderm lineage [34]. In periimplantation mouse embryos, Dab2 is exclusively expressed in extraembryonic endoderm, and its expression is not found in the embryo proper until after E8.5 [35]. Moreover, Dab2 is essential for the development of extraembryonic endoderm: Dab2 knockout in mice is early embryonic lethal due to the disorganization of the endoderm cells [35]. In this study, we investigated the differentiation of pluripotent mouse ES cells that are modified by the deletion of either GATA4, GATA5, or GATA6 genes. We determined whether the deficiency of an individual GATA factor altered the differentiating lineage of the ES cells and characterized the gene expression profiles of the differentiated cells by expression microarray analysis. The purpose of the experiments was to explore approaches to alter the lineage determination and differentiation of ES cells in vitro.

## 2. Materials and Methods

### 2.1. Reagents

All-trans retinoic acid was purchased from Sigma Aldrich (Milwaukee, WI). Tissue culture flasks (Falcon), media, trypsin, and 100X antibiotic-antimycotic solution (Cellgro, Mediatech, Inc) were purchased from Fisher Scientific Inc (Springfield, NJ). Trizol and Lipofectamine 2000 were purchased from Invitrogen (Carlsbad, CA). Leukemia Inhibitory Factor (LIF) was purchased from Chemicon (Temecula, CA). ES cell medium and serum were prepared by Fox Chase Cancer Center Tissue Culture Facility. For immunodetection, Super Signal West Dura Extended Duration Substrate (PIERCE, Rockford, IL) was used. Primary antibodies including rabbit anti-GATA4, goat anti-GATA5, goat anti-GATA6, and mouse anti-Oct-3/4 were purchased from Santa Cruz Biotechnology, Inc. (Santa Cruz, CA). Anti-Vimentin and anti-actin antibodies are purchased from Sigma Aldrich (Milwaukee, WI). Custom rabbit anti GATA-6 was also used for western blotting and immunofluorescence experiments. Alexa Fluor 488 and 596 conjugated secondary antibodies and DAPI nuclear counter staining dye were purchased from Invitrogen/Molecular Probes (Eugene, Oregon).

### 2.2. Cell Culture

Mouse ES cells were cultured on gelatin-coated tissue culture plates in ES cell media containing HEPES (6 g/l), 1X antibiotic-antimycotic, *β*-mercaptoethanol (0.14 *μ*M), 15% heat inactivated FBS, and LIF (1,000 U/mL). The plates were precoated overnight at 4°C with sterile gelatin solution (0.1%) and then washed three times with PBS prior to use.

RW-4 mouse ES cells and derived homozygous knockout cells of the GATA4 (−/−) [20], GATA5 (−/−), and GATA6 (−/−) [22] genotypes were maintained according to standard protocol. Prior to experiments, the ES cells were seeded on gelatin-coated plates or slide chambers in ES cell medium with LIF and without feeder cells. Cell differentiation was induced by treating with 1 *μ*M retinoic acid for four days. Alternatively, the cells were cultured on petri dishes to allow aggregation and formation of embryoid bodies for 4 days in ES cell medium in the absence of LIF.

### 2.3. Immunofluorescence Microscopy

Briefly, ES cells were seeded on gelatin-coated glass coverslips in 6-well dishes. Cells were washed twice with PBS at room temperature, fixed with 4% paraformaldehyde for 15 minutes, and permeabilized with 0.5% Triton X-100 for 5 minutes. Then the cells were washed three times with PBS, blocked with 3% BSA in PBS containing 0.1% Tween-20 for 30 minutes, and incubated for 1 hour at 37°C with primary antibodies diluted 1 : 200 in 1% BSA in PBS containing 0.1% Tween-20. GATA factor expression and localization was detected using AlexaFluor 488-conjugated (green fluorescence) or AlexaFluor 546-conjugated (red fluorescence) secondary antibodies. The cells were incubated in DAPI (blue color) solution for 3 minutes at room temperature to stain the nucleus. Cells were washed three times, then mounted and sealed in antifade reagent (Invitrogen/Molecular Probes). Stainings were viewed with 60X objective lens on a Nikon Eclipse TE 300 microscope linked to a Roper Scientific photometrics 12-bit range camera. Images were acquired using MetaVue software and merged using Adobe Photoshop software.

### 2.4. Electrophoretic Mobility Shift Assay (EMSA)

To analyze the binding ability of GATA transcription factors on the mouse Dab2 promoter, ES cells were differentiated with retinoic acid (1 *μ*M) for 4 days. Pellets of ES cells were collected and the cytoplasmic extract and nuclear extract were prepared according to published protocols [34]. Briefly, to isolate cytoplasmic extracts, cell pellets were incubated for 10 minutes on ice in 200 *μ*L of Buffer A containing HEPES (10 mM, pH 7.9), MgCl_2_ (1.5 mM), KCl (10 mM), DTT (0.5 mM), PMSF (0.2 mM), and a protease inhibitor cocktail (1X). Cell membranes were then ruptured with a pellet pestle (Fisher Scientifics) and the lysates were centrifuged at 10,000 rpm for 5 minutes to separate the cytoplasmic extracts from the nuclear pellets. To isolate nuclear extracts, nuclear pellets were incubated 30 minutes on ice in 50 *μ*L of buffer B containing HEPES (20 mM, pH 7.9), MgCl_2_ (1.5 mM), NaCl (420 mM), EDTA (0.2 mM), DTT (0.5 mM), PMSF (0.5 mM), protease inhibitor cocktail (1X), and glycerol (25%). Nuclear pellets were agitated every 5 minutes. After 30 minutes the lysates were centrifuged at 10,000 rpm for 5 minutes. The protein concentration of the supernatants (nuclear extracts) was determined using the Bio-Rad DC Protein assay and aliquots of the supernates were stored at −80°C until use. Oligonucleotide probes were end-labeled with 32P and T4 polynucleotide kinase, purified over a G25 spin column, and quantitated by liquid scintillation counting. The binding assay was performed as described previously [42]. The binding reaction contained HEPES-KOH (15 mM, pH 7.9), MgCl_2_ (1.5 mM), KCl (40 mM), DTT (0.5 mM), and glycerol (10%), 2 *μ*g Poly(dI-dC), nuclear extracts (10 *μ*g), and 20,000 cpm radiolabeled probe in a total volume of 20 *μ*l. The binding reaction mixture was incubated on ice for 30 minutes. For super-shift assays, antibodies to GATA4, GATA5, and GATA6 were added to the corresponding reaction and incubated on ice for 1 hour. Aliquots (20 *μ*l) of the binding reaction were loaded on 5% nondenaturing polyacrylamide gels and run at 120 V and 4°C for 3 hours in 0.5 X TBE running buffer using a Vertical Gel Electrophoresis Apparatus (Model V15-17, GibcoBRL) to separate bound and unbound oligonucleotides. Gels were dried for 1 hour and exposed to X-ray film (Fuji) for 18 hours at −80°C. We identified and tested four GATA binding sites located upstream of the ATG site of the mouse Dab2 gene (−1904, −1926, −3943, −3894 bp). The GATA-binding oligonucleotides from region −1904 bp (PI) and −1926 bp (PII) were most potent for GATA complex formation in nuclear extracts from ES cells treated with retinoic acid or from rat cardiomyocytes. The GATA-binding oligonucleotide sequences are as follows:

  PI, 5′-ACACATTTTGATAATAATCTTT-3′;
  PII, 5′- CAACTATATAGATAAAGACAAAGG-3′.

The unlabelled PI probe and an SP1 probe were used at 300-fold excess as specific and nonspecific competitors, respectively [42]. To test the specificity of the GATA binding sequence, we also mutated the “GAT” of the consensus sequence to CGC, to synthesize m1PI′ and m2PI′ for competition assays. The sequences of these probes are shown in Figure 6.

### 2.5. Reverse Transcription-Real-Time Polymerase Chain Reaction Analysis

Total RNA was extracted from ES cells mock treated (media + DMSO) or treated with retinoic acid for 4 days using Trizol reagent according to the manufacturer's protocol. RNA was purified with Qiagen Rneasy mini kit to remove contaminants. Potential contaminating DNA was removed using TURBO DNA-free kit (Ambion). RNA was quantified using the Agilent 2100 BioAnalyzer in combination with an RNA 6000 Nano LabChip. RNA was reverse-transcribed (RT) using the M-MLV reverse transcriptase (Ambion) and a mixture of anchored oligo dT and random decamers. Two different amounts of input RNA (100 and 20 ng) were used to monitor the linearity of the RT reaction and the efficiency of PCR. Real-time PCR “Assay-on-demand” (Applied Biosystems) Taqman assays were run using an ABI 7900 HT instrument. The information on the genes analyzed by real-time RT-PCR is listed in Table 2.

### 2.6. DNA Expression Array

Gene expression profiles were analyzed to compare undifferentiated and retinoic acid differentiated ES cells of wildtype and GATA-deficient genotypes using a mouse 32 K oligochip printed by the Microarray Facility of Fox Chase Cancer Center [43]. Total RNA was isolated from 60%–80% confluent ES cell cultures using the guanidinium/isothiocyanate/phenol/chloroform method. Total RNA was DNase treated using “DNA free” kit (Ambion, Austin, TX) according to the manufacturer's specifications. Fifteen micrograms of this DNase-treated RNA were reverse transcribed and amino allyl dUTP was incorporated in a reaction containing 500 ng oligo (dT) primers, 1x first strand buffer (Invitrogen, Carlsbad, CA), 0.01 M DTT, 500 *μ*M each of dATP, dCTP, dGTP, and dTTP/aadUTP (2 : 3 ratio), 40 Units of rRNasin (Promega, Madison, WI), and 200 Units of SuperScript II reverse transcriptase (Invitrogen, Carlsbad, CA). After brief denaturation and annealing of the primers at 70°C for 8 minutes, the reaction was incubated at 42°C for 2 hours, followed by alkali hydrolysis of RNA and cDNA purification using Microcon-30 columns (Millipore, Bedford, MA), and then labeled with either Cy3- or Cy5-dyes by a coupling reaction using FluoroLinkTM monofunctional dyes (Amersham Pharmacia Biotech, Piscataway, NJ) according to the manufacturer's specifications. Probes were then purified using StrataPrep PCR Purification Kit (Stratagene, La Jolla, CA). Pairs of the samples (one labeled with Cy3 and one with Cy5) were combined, denatured, and preannealed in the presence of 10 *μ*g of Cot-1 DNA (Invitrogen, Carlsbad, CA) and 10 *μ*g of poly-dA DNA. Hybridization and washes were performed as previously described [43]. The procedure was repeated for each sample, except that the dyes used to label the RNAs were reversed. Furthermore, for each time point the hybridizations were repeated using an independent source of RNA. Intensity extraction and spot quality characteristics were performed as previously described [43].

The microarray data was then analyzed using GeneSight 3 software (BioDiscovery, Inc., Marina Del Rey, CA). The data was (i) corrected for background by subtraction of the local group median, (ii) normalized using a piecewise linear normalization with 5 bins, typically greater than 1000 data points per bin, and (iii) limited to a minimum expression level equal to an estimate of the minimum background. The data was then converted to log_2_ values, and the mean and standard deviation determined for each intensity ratio by combining “dye-flip” replicates. At least two “dye-flip” experiments were performed for each sample. The mean and the coefficient of variance were calculated for these values and used for statistical analysis and clustering. Differential expression of individual genes was determined by confidence analysis [44] and maximum likelihood analysis [45] to obtain a final list of candidates at a >99% confidence level. The data was also analyzed using hierarchical clustering [46] with a Euclidean distance metric.

## 3. Results

### 3.1. Endoderm Lineage Differentiation of Wildtype and GATA Deficient Embryonic Stem Cells Induced by Aggregation and/or Retinoic Acid

**Figure 1:**
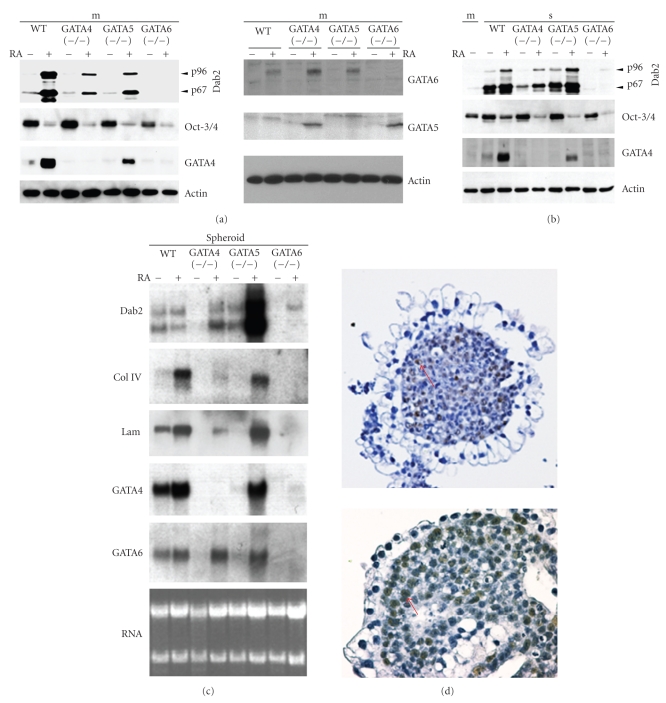
Endoderm lineage differentiation of ES cells in vitro. Approximately 1 × 10^6^ ES cells of wildtype or deficient in one of GATA factor were seeded on 100 mm plates as a monolayer culture (m) or cultured in suspension to allow cell aggregation to form spheroids (s). The cells were also treated with 1 *μ*M retinoic acid (RA) or DMSO control. Following a 4-day culture period, cell lysates from monolayer (a) or from spheroids (b) were prepared for Western blotting analysis. (c) mRNA was prepared for Northern blot analysis. (d) Preservation of Oct-3/4 protein in embryoid bodies: embryonic stem cells were cultured in medium lacking LIF in suspension to allow the formation of cell aggregates. The embryoid bodies from a 4-day suspension culture were fixed in formalin, embedded in paraffin, sectioned, and immunostained for Oct-3/4 protein. Representative stainings of two embryoid bodies are shown (left panel, 40×, and right panel, 200×).

We first compared general markers for endoderm differentiation of wildtype and GATA deficient ES cells following retinoic acid treatment and/or aggregation, by Western blot (Figures 1(a) and 1(b)) and Northern blot (Figure 1(c)) analyses. Induction of Dab2, collagen IV, and laminin was used as markers for commitment to the extraembryonic endoderm lineage. As shown by Western blot analysis (Figures 1(a) and 1(b)), wildtype ES cells differentiated into endoderm cells after either retinoic acid treatment or aggregation, as indicated by the expression of Dab2. GATA4 deficient ES cells, however, did not respond to cell aggregation; little Dab2 was induced (Figure 1(b)), but did undergo endoderm differentiation when treated with retinoic acid in either monolayer or cell aggregates (Figures 1(a) and 1(b)), consistent with previous reports [25]. Notably, GATA4 (−/−) ES cells showed a quantitatively reduced endoderm differentiation compared to wildtype cells in response to either retinoic acid or aggregation. GATA5-deficient ES cells in monolayer culture exhibited a reduced response to retinoic acid for Dab2 induction/endoderm differentiation. Nevertheless, aggregation with or without the presence of retinoic acid induced endoderm differentiation (Figure 1(b)). Neither Dab2 nor GATA4 was induced by aggregation and/or retinoic acid treatment in the GATA6 (−/−) cells. Thus, GATA6 is critically required for endoderm differentiation of ES cells induced either by retinoic acid treatment and/or aggregation, consistent with previous studies [22, 23, 25]. In the absence of GATA6, GATA5 is induced by retinoic acid (Figure 1(a)) but is insufficient to induce primitive endoderm lineage differentiation.

**Figure 2:**
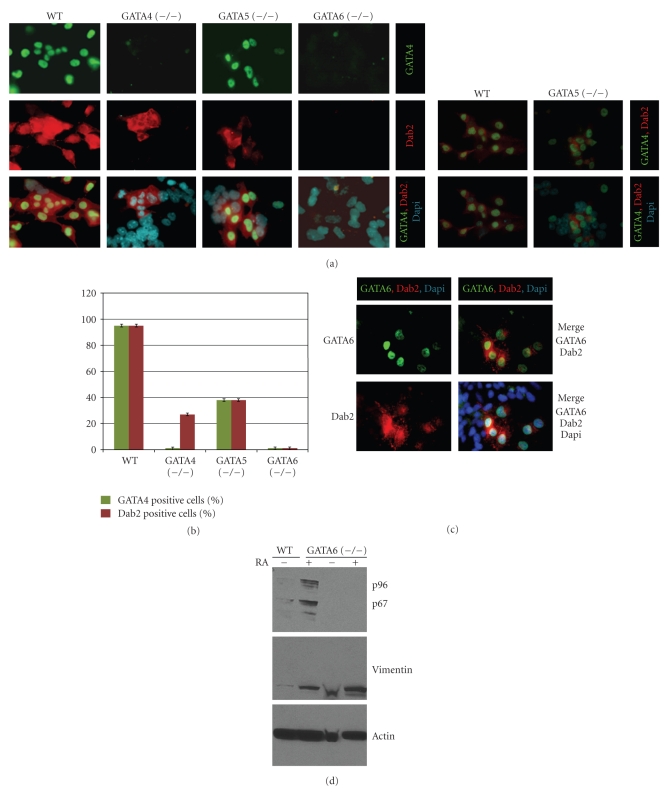
Retinoic acid-induced endoderm differentiation of GATA-deficient ES cells in vitro: ES cells of wildtype, GATA4 (−/−), GATA5 (−/−), or GATA6 (−/−) genotypes in monolayers were treated with or without retinoic acid (1 *μ*M) for 4 days. Cells expressing Dab2 and GATA4 were detected following indirect immunofluorescence staining and counting under fluorescence microscopy. (a) Dab2 (red) and GATA4 (green) were detected under fluorescence microscopy with DAPI (blue) used for nuclear counterstaining. The percentage of Dab2 and GATA4 positive cells was determined by counting an average of 5 fields of cells. (b) The percentages of ES cells expressing GATA4 and Dab2 are presented in histogram. (c) In GATA4 (−/−) the expression of Dab2 correlates with the expression of GATA6. (d) Western blotting showing the absence of endoderm marker Dab2 and expression of mesoderm marker vimentin in GATA6 (−/−) ES cells.

The reduction of Oct-3/4 was used as an indicator of the loss of pluripotency and differentiation of ES cells. Oct-3/4 protein levels were reduced similarly in wildtype and all GATA-deficient (including GATA6-deficient) ES cells upon retinoic acid treatment, indicating that retinoic acid induced the differentiation of ES cells irrespective of any GATA factors. Thus, retinoic acid induces the differentiation of GATA6 (−/−) ES cells to a lineage other than primitive endoderm. Without retinoic acid treatment, the Oct-3/4 level was not reduced upon cell aggregation-induced differentiation in wildtype or GATA-deficient ES cells (Figure 1(b)), suggesting that the formation of embryoid bodies can preserve pluripotency in a subset of ES cells. Immunostaining showed that the Oct-3/4-positive cells locate in the interior of the embryoid bodies (Figure 1(d), an example of a Oct-3/4-positive cell is indicated by an arrow). Both GATA5 and GATA6 proteins were difficult to detect by Western blot in cell aggregates (not shown), likely because of the smaller number of the cells undergoing primitive endoderm differentiation. The expressions of laminin and collagen IV are also difficult to measure by western blotting because they are secreted proteins. Thus, we used potentially more sensitive assays such as Northern blot (Figure 1(c)) to measure gene expression in spheroids. Northern blotting is also able to measure the expression of the secreted proteins laminin and collagen IV, which form the basement membrane in spheroids. Curiously, Dab2 mRNA was induced in the aggregated GATA5 (−/−) ES cells, but GATA4, laminin, and collagen IV were not significantly induced (Figure 1(c)). Following retinoic acid treatment, GATA5 (−/−) ES cells exhibited an exaggerated response to retinoic acid and induced the mRNA of Dab2, GATA4, and GATA6 (Figure 1(c)). The northern blot results show a disparity between mRNA (Figure 1(c)) and protein (Figure 1(b)) levels of Dab2 and GATA4 in these cells. Protein and mRNA were measured from the same preparation of cells to show reproducibility, and these results were obtained repeatedly in three experiments. A likely interpretation of these data is that GATA5 may have a potential role in regulating the translation of these mRNAs into proteins. Either a variation in gene expression level in the wildtype and GATA-deficient cells or a difference in the percentage of cells that undergo differentiation may account for the observed differences in the expression of endoderm markers determined by Western and Northern blot analysis. This question was addressed by immunofluorescence microscopy to detect the expression of GATA4 and Dab2 in individual cells (Figure 2(a)). When wildtype ES cells were exposed to retinoic acid, more than 95% of the cells differentiated into the GATA4 and Dab2 positive extraembryonic primitive endoderm cells. However, retinoic acid-induced extraembryonic endoderm differentiation was reduced in ES cells that lacked any GATA factor. The percentages of cells that differentiated into primitive endoderm lineage (Dab2-positive) were GATA-4 (−/−), 27%; GATA-5 (−/−), 38%, and GATA-6 (−/−), <1% (Figure 2(b)). The percentages of Dab2 and GATA4 positive cells for WT, GATA-4 (−/−), GATA-5 (−/−), and GATA-6 (−/−) were determined by counting 100 cells in 5 microscopy fields and the averages are presented in histogram (Figure 2(b)). In WT ES cells the majority of cells expressing GATA4 express also Dab2. Since expression of Dab2 is required for extraembryonic endoderm development [35], the absence of Dab2 is a likely indicator of the lack of endoderm differentiation. In GATA-4 (−/−) ES cells the expression of Dab2 is confined to a subset of cells and correlates with the expression of GATA6 (Figure 2(c)). GATA6 (−/−) ES cells are negative for Dab2 but express vimentin which is stem cell marker for mesoderm differentiation (Figure 2(d)). Thus, the main differentiation route following retinoic acid treatment of wildtype ES cells is the extraembryonic endoderm lineage. However, the absence of a GATA factor reduces extraembryonic endoderm differentiation and promotes the ES cells to adopt alternative fates.

### 3.2. Requirement of GATA5 in Aggregation-Induced Expression and Formation of Basement Membrane in Embryoid Bodies

**Figure 3:**
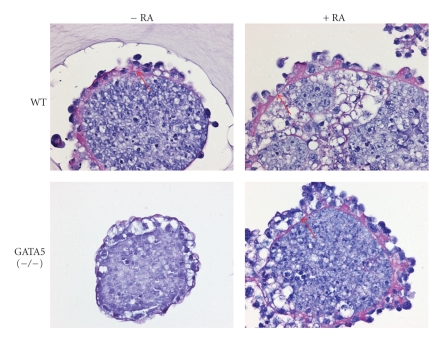
Primitive endoderm differentiation and the absence of the basement membrane in GATA5-deficient embryoid bodies. Wildtype and GATA5 (−/−) ES cells were cultured in medium lacking LIF in suspension to allow the formation of cell aggregates on Petri dishes, with or without retinoic acid (1 *μ*M) for 4 days. The embryoid bodies from a 4-day suspension culture were fixed, embedded in paraffin, sectioned, and subjected to PAS staining to detect the basement membrane. Representative stainings are shown (200×). Spheroids of GATA4 (−/−) and GATA6 (−/−) ES cells were previously published [25] and showed that endoderm formation is impaired in GATA4 (−/−) ES cell spheroids without RA. Endoderm formation in GATA4 (−/−) ES cell spheroids can be restored by treatment with RA for 4 days. Spheroid of GATA6 (−/−) ES cells lacked endoderm formation that cannot be restored with RA treatment [25].

One peculiar observation is that embryoid bodies derived from GATA5 (−/−) ES cells were Dab2 positive but lacked the expression of laminin and collagen IV (Figure 1(c)). We examined the morphology and the presence or absence of a basement membrane in the embryoid bodies formed by PAS staining, which detects the glycoproteins of the basement membrane [36, 37]. Embryoid bodies derived from GATA5 (−/−) cells exhibited an endoderm outer layer that consisted of vacuous (visceral endoderm) cells. PAS staining was negative in GATA5 (−/−) embryoid bodies, indicating the absence of a basement membrane, while embryoid bodies formed from wildtype ES cells showed a distinctive basement membrane underneath a layer of parietal endoderm-like cells (Figure 3, arrow). However, basement membranes were present in both wildtype and GATA5 (−/−) embryoid bodies when treated with retinoic acid. Thus, GATA5 is required for aggregation-induced expression of laminin and collagen IV and the formation of a basement membrane. Additionally, basement membrane formation is not essential for the formation of the visceral endoderm epithelium in embryoid bodies [38, 39]. This data suggests that GATA5 may be required for aggregation-induced differentiation of ES cells into parietal endoderm cells, which are active in the production of basement membranes and formation of Reichert's membrane [40]. Consistent with this possibility, the wildtype ES cells form spheroids covered with surface epithelial cells that resemble parietal endoderm (flat cells), and GATA5 (−/−) ES cells form spheroids covered with epithelial cells that resemble vacuous visceral endoderm cells (Figure 3).

### 3.3. Binding of GATA Factors to the Dab2 Promoter

**Figure 4:**
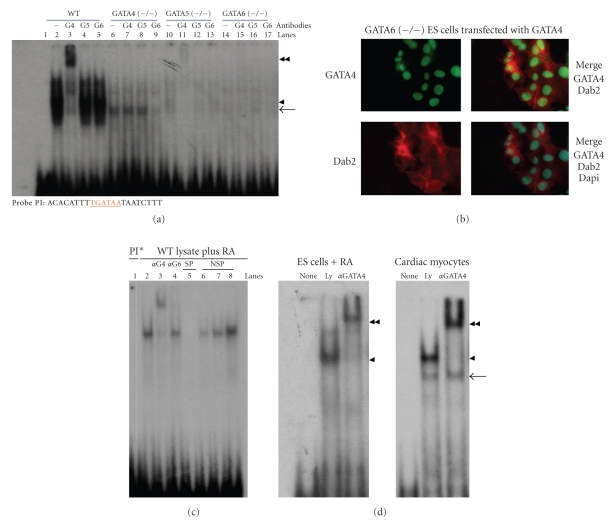
GATA-binding activity in retinoic acid-induced endoderm differentiation of wildtype and GATA-deficient ES cells. Wild type ES cells and those homozygous deficient in GATA4, GATA5, or GATA6 were treated with retinoic acid (1 *μ*M) for 4 days. Nuclear extracts of the ES cells were used for EMSA and supershifted with antibodies specific for each GATA factor. (a) For EMSA, the labeled Dab2-PI GATA binding probes were incubated with nuclear extracts from ES cells treated with retinoic acid (1 *μ*M) for 4 days. GATA6 (arrow) and GATA4 (arrowhead) containing complexes are indicated. The supershifted GATA4-containing complexes are indicated by a double-arrowhead. (b) GATA6 (−/−) ES cells in monolayers were transfected with GATA4 and immunofluorescence stainings of Dab2 (red) and GATA4 (green) were observed by fluorescence microscopy. DAPI (blue) was used for nuclear counter staining. (c) The binding to the PI probe is compared between nuclear extracts from retinoic acid-treated ES cells and cardiomyocytes. GATA4-containing (arrowhead), anti-GATA4 supershifted (double arrowhead), and GATA6-containing (arrow) complexes are indicated. Self competition (SP) assay was performed with nonradioactive PI probe and nonspecific competitions were performed with m1pI: PI probe with mutation in GATA binding site 1 (lane 6); with m2pI: PI probe with mutation in GATA binding site 2 (lane 7), and m1pI/m2 m2pI: probe PI with mutations in both GATA binding sites (lane 8). *α*G4 and *α*G6: antibodies against GATA4 and GATA6; SP: self competition with cold PI; NSP: nonspecific competition.

We next investigated the promoter binding activity of GATA factors in nuclear extracts of wildtype and GATA-deficient ES cells [41]. We identified and tested four GATA binding sites located upstream of the ATG site of the mouse Dab2 gene (−1904, −1926, −3943, −3894 bp). The GATA-binding oligonucleotides from region −1904 bp (PI) and −1926 bp (PII) were most potent for GATA complex formation in nuclear extracts from ES cells treated with RA or from rat cardiomyocytes. We used a predicted GATA-binding site from the mouse Dab2 promoter (referred to as Dab2-PI site, at −1904 bp) to perform electrophoretic mobility shift assays (EMSA) using nuclear extracts from retinoic acid-treated cells [42]. As shown in Figure 4(a), wildtype ES cells possessed the highest GATA binding activity. Most of the oligo-binding complexes (arrowhead) were super-shifted by antibodies to GATA4 (doublearrowhead) but not by antibodies to GATA5 or GATA6 (Figure 4(a)). In GATA4 (−/−) cells, a complex (arrow, Figure 4(a)) was seen that could be eliminated by antibodies to GATA6, but not by antibodies to GATA4 or GATA5. Comparing to wildtype ES cells, the loss of any GATA factor greatly reduced binding activity to the GATA site on Dab2 promoter probes, suggesting that optimal binding requires the presence of all the three GATA factors. In GATA5 (−/−) cells the number of cells expressing GATA4 and Dab2 was low; thus, only a weak GATA-4-containing complex was shifted by anti-GATA4 antibodies (Figure 4(a)). GATA6 (−/−) ES cells do not express GATA4 or Dab2 and no GATA binding activity to Dab2 promoter was observed; but transfection of GATA4 in GATA6 (−/−) ES cells induces Dab2 (red) and GATA4 (green) expression as observed by fluorescence microscopy, and DAPI (blue) was used for nuclear counter staining (Figure 4(b)). This data suggests that GATA4 is required for optimal expression of Dab2 and differentiation of ES cells into primitive endoderm. All the binding activities observed seem to be specific to the probe, since the inclusion of unlabeled specific probe PI in excess of 300-fold inhibited the binding of GATA factors to radiolabel PI probe of Dab2 promoter (Figure 4(c)). Also, the binding is specific to the GATA binding sites since introduction of mutations in either forward (m1PI) or reverse (m2PI) GATA binding site in the sequence abolished the ability of the unlabeled probe to compete with labeled PI (Figure 4(c)). EMSA was also performed with another predicted GATA-binding site from the Dab2 promoter (referred to as Dab2-PII site, at −1926 bp). Similar results were obtained from Dab2-PII probe as those using the Dab2-PI probe, suggesting that the GATA-binding properties of ES cells are similar to both GATA-binding sites (data not shown).

The EMSA patterns from ES cells were also compared to binding activity from rat cardiomyocytes using the same probe (Figure 4(d)). Nuclear extracts from either ES cells or cardiomyocytes contain GATA4-containing complexes (arrowhead) that can be supershifted with antibodies to GATA4 (double arrowhead), and cardiomyocytes contain more of a GATA4-negative complex (arrow). Thus, GATA binding activities differ only subtly between the retinoic acid-differentiated ES cells and cardiac myocytes, suggesting that there are some differences in the presence of cofactors in the two different cell types.

### 3.4. Comparison of Gene Expression Profiles in Endoderm Differentiation of Wildtype and GATA Deficient ES Cells in Monolayer Culture

To further characterize the influence of the deficiency of each GATA factor on the differentiation of ES cells, we applied a cDNA microarray analysis [43] to compare gene expression profiles of wildtype, GATA4 (−/−), GATA5 (−/−), and GATA6 (−/−) ES cells following retinoic acid-induced differentiation of these ES cells cultured as monolayers. The data has been submitted to the National Center for Biotechnology Information (NCBI) Gene Expression Omnibus (GEO) repository (A link will be found at http://www.ncbi.nlm.nih.gov/projects/geo/query/acc.cgi?acc=GPL4486, and the link will be activated upon publication of the manuscript). 

**Figure 5:**
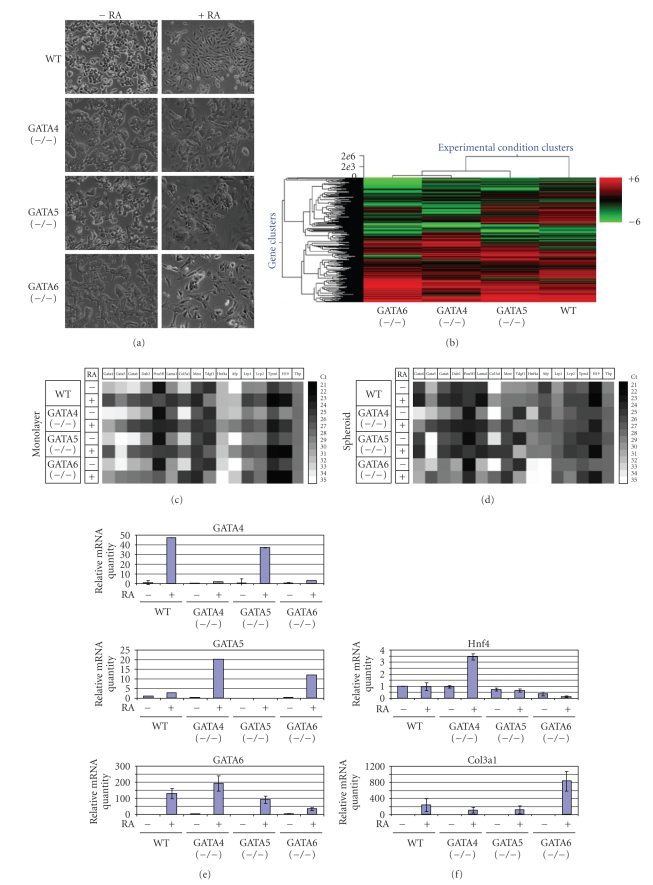

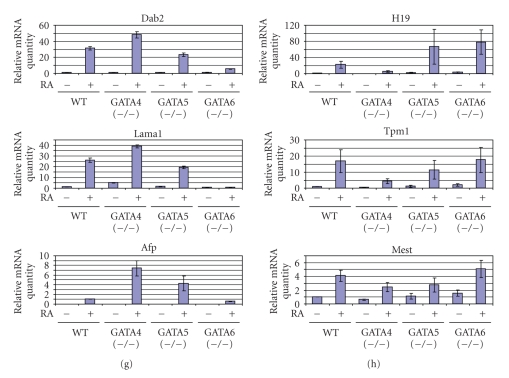
Expression array analysis and verification by quantitative RT-PCR of retinoic acid-induced endoderm differentiation of GATA-deficient ES cells. Wild type embryonic stem cells and those homozygous deficient in GATA4, GATA5, or GATA6 were cultured as monolayers and treated with the DMSO carrier as a control or retinoic acid (1 *μ*M) for 4 days. (a) Representative morphology of the cells with or without retinoic acid. (b) The cells were analyzed by cDNA expression microarray comparing with or without retinoic acid. The results were analyzed by hierarchical clustering. Individual colored rows represent change in expression following retinoic acid treatment of a single gene/sequence tag. Red rows indicate an increase in expression and green rows indicate a decrease in expression, as shown by the color scale bar. (c)–(h) Verification of expression of a panel of selected genes by quantitative RT-PCR Relative gene expression changes with or without retinoic acid in monolayer cells (c) and spheroids (d) are presented as “Heat-Maps”. The figure displays grey scale shades representing the Ct values. Ct (cycle threshold) is the number of PCR cycles at which the fluorescence reaches a significant level above the baseline, given that the higher the starting copy number of the nucleic acid target, the sooner fluorescence increases. The relative levels of a particular transcript between samples can be calculated using the equation: relative quantity = 2 − ∆Ct. The amplification of TBP shows similar amounts of template in all samples. (e)–(h) values that represented relative mRNA levels of the monolayer ES cells are shown and compared. The mRNA values of undifferentiated ES cells are defined as “1” for comparison. The detection of a real-time RT-PCR signal in a specific GATA transcript in the ES cells that were homozygous knockout of that GATA gene is likely due to the presence of transcripts from the mutant/inactive GATA locus.

**Figure 6:**
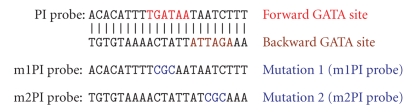


Examination of individual genes, such as Dab2, laminin, collagen, and each GATA factor showed that the changes are consistent with Western and Northern blotting analysis (Table 1) with exception of collagen IV which shows some difference between the ES cells monolayer cDNA array and the spheroid northern blot. For most of the other genes the cDNA array approach verified the northern blotting and the western blotting results. The morphology of undifferentiated wildtype or GATA-deficient ES cells is similar in absence of retinoic acid. Treatment with retinoic acid, however, induced unique morphological changes in each cell type (Figure 5(a)), suggesting that the differential expression of GATA factors influences cell properties including morphology.

The expression profiles of the wildtype and GATA knockout ES cells induced by retinoic acid were compared by significance analysis of microarrays (SAM) and hierarchical clustering (Figure 5(b)) [44–46]. Visual observation indicates that the effect of GATA4, GATA5, or GATA6 deficiency on retinoic acid-induce changes in gene expression is profound and the deletion of each GATA factor shows a drastically altered gene expression profile (Figure 5(b)). In experiments comparing signals of RNA from various preparations (2 to 4 preparations for each cell types) of the same cell type, the expression profiles were similar, indicating that the observed differences between cell types were not due to experimental variation but reflected expression differences associated with the genotypes. Sorting by mathematical modeling [46] indicates that expression profiles are more similar between wildtype and GATA5 (−/−) ES cells than either GATA4 (−/−) or GATA6 (−/−) ES cells (Figure 5(b)). Thus, GATA4, GATA5, and GATA6 can all dramatically modify gene expression profiles of ES cells following retinoic acid treatment. Although many changes in gene expression are apparently associated with the endoderm lineage, most of these changes (that occur in GATA4 (−/−) and GATA5 (−/−) ES cells) are not critical for the function of the endoderm cells, and only a few critical genes, such as Dab2, may be required for endoderm formation in early embryonic development. The cDNA arrays show that GATA6 (−/−) ES cells differentiate toward mesoderm lineage with expression of mesoderm markers (mesoderm transcript, collagen III alpha) and cardiac marker (tropomyosin alpha1).

### 3.5. Verification of a Panel of Markers Identified from CDNA Microarray Analysis

We selected a panel of genes identified from the expression array experiments with large fold changes (either upregulated or downregulated) for further verification (Table 2). Several lineage markers with known importance in ES cell differentiation, which were also identified to have significant changes in the expression array, were included for comparison. The expression of these genes was analyzed by quantitative real-time PCR, and the result is presented as “Heat Maps” (Figure 5(c)). In this experiment, we also investigated the expression pattern of this panel of genes in embryoid bodies treated with or without retinoic acid (Figure 5(d)).

By comparing gene expression in monolayer (Figure 5(c)) and spheroids (Figure 5(d)), aggregation-induced genes were found to include GATA4, GATA6, Dab2, Laminin (lama1), Afp, H19, and Lrp2, in wildtype ES cells in the absence of retinoic acid. Aggregation is able to reduce expression of Tdgf1 (cripto) and Oct-3/4 (pou5f1) in wildtype ES cells in the absence of retinoic acid. Expression of GATA5, Col3*α*1, MEST, and Lrp1 is dependent on retinoic acid in the wildtype ES cells. Some unique and subtle differences in gene expression profiles in response to retinoic acid versus aggregation are observed in wildtype and GATA-deficient ES cells (Figures 5(c) and 5(d)). For example, in monolayer culture of GATA6 (−/−) ES cells, retinoic acid induced higher expression of Col3*α*1, MEST, and Tpm1 than in spheroid culture. 

Several points from the analysis of the expression profiles (Figure 5) can be noted. First, there is interregulation between GATA factor expression (Figure 5(e)). Retinoic acid-induced GATA4 expression depends on GATA6 but not on GATA5; the induction of GATA5 expression by retinoic acid is enhanced by the deletion of either GATA4 or GATA6; deletion of either GATA4 or GATA5 does not significantly alter retinoic acid-induced GATA6 expression. 

HNF4 was strongly upregulated by retinoic acid in GATA4 (−/−) ES cells in correlation with the upregulation of GATA6 (Figure 5(f)). In the absence of GATA6, mesoderm lineage seems to be favored, as indicated by the expression of collagen 3alpha1 (Figure 5(f)), which has been reported to be specifically expressed in embryonic mesoderm, sclerotomes, dermatomes, and in forming connective tissues [47]. GATA6 is required for endoderm lineage differentiation, as indicated by the reduced expression of endoderm markers such as Dab2, laminin, and Afp in both the absence or presence of retinoic acid (Figure 5(g)).

Other GATA6-independent (but GATA4-dependent) genes include fetal liver transcript H19 [48], cardiac marker tropomyosin 1 alpha (Tpm1), and Mest, the mesoderm specific transcript [49, 50] (Figure 5(h)). In general, deletion of an individual GATA factor seems to promote alternative lineages of ES cell differentiation.

## 4. Discussion

We investigated the in vitro differentiation of murine ES cells with a deficiency of either GATA4, GATA5, or GATA6 and compared them to wildtype ES cells. The rationale for the experiments was that GATA factors are involved in the early step of cell lineage determination of the pluripotent cells of the inner cell mass, the in vivo equivalents of ES cells, and thus alterations of GATA factors might influence ES cell lineage determination.

The inability of GATA6 (−/−) ES cells to undergo extraembryonic endoderm differentiation is consistent with the finding that GATA6 deletion results in defect in endoderm development and early embryonic lethality [22, 23]. Although deletion of either GATA4 or GATA5 reduces but not impairs endoderm lineage differentiation of ES cells, the loss of either GATA4 or GATA5 does not block extraembryonic endoderm development in mouse early embryos [30, 51]. Nevertheless, the GATA4 null endoderm is defective in its role in cardiac induction [19, 52]. Likely, the requirement of GATA4 and GATA5 in extraembryonic induction observed in vitro can be compensated in vivo, but the GATA factors may contribute to other uncharacterized extraembryonic endoderm function. 

Wildtype ES cells express endoderm markers such as Dab2, GATA4, GATA6, laminin, and collagen IV following differentiation with retinoic acid or aggregation. Expression of GATA5 is not observed in wildtype ES treated with retinoic acid but is observed in GATA4 (−/−) ES or GATA6 (−/−) ES cells treated with retinoic acid. GATA4 (−/−) ES cells express liver markers HNF4 and GATA6 in vitro; indeed, study reported that GATA6 regulates HNF4 and is required for differentiation of visceral endoderm in the mouse embryo [22]. GATA6 (−/−) ES cells express mesoderm and cardiac markers (mesoderm transcript, collagen 3 alpha1 and tropomyosin 1 alpha), while GATA5 (−/−) cells seem to have regulatory roles in the expression of endoderm marker proteins. We observed that in GATA5 (−/−) ES cells, the mRNA and protein levels of several endoderm markers including Dab2 and GATA4 are disassociated (Figure 1). When stimulated by retinoic acid, endoderm markers are highly induced at the mRNA level in spheroids from GATA5 (−/−) ES cells, to a level much greater than that in wildtype ES cells. However, judging from the protein level, GATA5 (−/−) ES cells express less of these endoderm markers. Thus, GATA5 may have a role in the regulation of the translation of these mRNAs into protein. We also found that the cell-aggregation-induced expression of basement membrane components requires GATA5. The ability to produce proper basement membrane is thought to be important for early embryogenesis [36–39]. However, GATA5 deficiency does not appear to impact primitive endoderm formation in mouse embryonic development [30].

We found that GATA5 (−/−) ES cells exhibit only a subtle phenotype in the formation of embryoid bodies. Likely, the function of GATA5 in primitive endoderm development is redundant with GATA4 and GATA6. Alternatively, GATA5 may be needed for aggregation-induced ES cell differentiation into parietal endoderm lineage. The parietal endoderm cells express high levels of basement membrane components (laminin and collagen IV) and are responsible for producing basement membrane for the thick Reichert's membrane in the early embryos [8, 10]. Several observations from these experiments may have biological implications to establish regenerative therapy. Differentiation of ES cells could be directed to mesoderm formation by deletion of GATA6, which may improve the efficiency to generate cardiomyocytes derived from ES cells. Deletion of GATA4 in ES cells could direct to formation of liver cells in vitro. However, much more work is needed to improve the use of deletion of either GATA factor for regenerative therapy.

## 5. Conclusions

We found that the deletion of one GATA factor, either GATA4, GATA5, or GATA6, can drastically alter the gene expression profiles and lineage determination of ES cells induced to differentiate by retinoic acid. ES cells lacking a single GATA factor, either GATA4, GATA5, or GATA6, exhibit a unique pattern of gene expression profile when differentiated. Deletion of GATA6 terminates the differentiation of ES cells to endoderm but leads to mesoderm lineage differentiation. Normally, during in vitro differentiation, the majority of ES cells differentiate into primitive endoderm cells [25]. Thus, the deletion of GATA6 allows the selection of lineage other than yolk sac endoderm. This study demonstrates a potential approach in redirecting the lineage determination of ES cells in vitro by altering the expression of GATA factors.

## Figures and Tables

**Table 1:** Information on genes analyzed by real-time RT-PCR using Taqman assay.

Gene Symbol	NCBI Gene Reference	Target Exons	Context Sequence
Tbp	NM_013684,NM_013684	3	ATCCCAAGCGATTTGCTGCAGTCAT
Gata4	NM_008092,AF179424,U85046, M98339,AB075549	3	CGCCGCCTGTCCGCTTCCCGCCGGG
Gata5	NM_008093,U84725	1	AGGACCAGCTTCGTACCTGACTTCT
Gata6	NM_010258,AF179425	5	CTCAGGGGTAGGGGCATCAGTGATG
Dab2	NM_023118,U18869	1	TAGCTAGTCCGTGTACTTTGTGGGT
Pou5f1	NM_013633,X52437,M34381,BC068268	2	GCGTTCTCTTTGGAAAGGTGTTCAG
Afp	NM_007423,V00743,BC066206, AK075972, AK010934,AK076053,AK076197	3	GTGTTTAGAAAGCCAGCTATCTGTG
Mest	NM_008590,D16262,AK031718,AK032881,AK034949,BC006639	1	TCGCTTGCGCAGGATGAGAGAGTGG
Lpr2	AF197160,BC040788	11	CTATGCAGAGATGGACACTGAGCAA
Hnf4a	NM_008261,D29015,BC039220	8	ATGCTTCTCGGAGGGTCTGCCAGTG
Lrp1	NM_008512,X67469,AF367720	15	CCTGCTTGGCGAACCCATCCTACGT
Lama1	NM_008480,J04064	19	AAACTGCCGAGCCTGTGACTGCCAC
Col3a1	NM_009930,BC058724,M18933,AK029212, BC052398,AK013329,AK041115,AK048546,BC043089	4	GTGGCCAAAATTATTCTCCCCAATT
Tdgf1	NM_011562,M87321	15	AAGACTGGGGAAACAGAGTGGATTG
Tpm1	NM_024427,X64831,M22479,BC026720, AK002271,AK003175,AK032942,AK077713	1	CGGAGCAAGCAGCTGGAAGATGAGC
H19	X58196,BC025150	1	GGACTGGAGACTAGGCCAGGTCTCC

Tbp was used as normalization control.

Afp: alpha fetal protein; Col3a1: collagen III alpha 1; H19: fetal liver RNA transcript; Lama 1: laminin alpha 1 gene; Lrp: lipoprotein-related receptor protein; Mest: mesoderm-specific transcript; Pou5f1: Oct-3/4; Tdgf1: Teratocarcinoma derived growth factor 1 (cripto); Tbp: TATA box binding protein; Tpm1: tropomyosin 1, alpha.

**Table 2:** Limited list of genes whose expression is significantly altered following retinoic acid treatment of ES cells grow in monolayer cultures.

	WT	GATA4** (**−/−**)**	GATA5 **(**−/−**)**	GATA6** (**−/−**)**
*Dab2*	4.38, 3.77	2.00, 1.80	4.09, 3.24	0.05, 0.48
GATA4	3.32	0.22	2.58	0.91
GATA6	2.04, 2.74, 2.51, 2.38	2.15, 2.08	1.94	0.41, 0.01
*collagen IVa1*	2.58, 2.30	2.86, 2.73	2.94, 2.72	2.72, 3.00, 2.84
*collagen IVa2*	1.88	2.66	2.48	2.48, 3.03
*laminin alpha1*	2.52	2.59	3.79	0.20
*laminin beta1*	2.74, 2.65	2.13	3.05	2.01
*laminin gamma1*	2.27, 2.00	2.20	2.38, 2.94, 2.02	2.90
*keratin gene 8*	3.20, 3.005, 2.301	2.59, 2.2, 2.17	2.27, 1.61	3.08, 3.05, 2.68, 1.99
*procollagen III*	0.87	0.48	1.07	3.96
*lrp1*	2.55	1.76	2.90, 2.35	0.15, 0.27
*lrp2*	2.28, 2.15	1.83	2.37	0.09, 0.65
*Talin*	2.52	2.87	2.93	3.62
fetal liver H19	2.20, 2.65, 2.35	2.65, 2.38	3.86, 3.80, 3.68	3.82, 3.80
*igf2*	2.29	3.26	2.66	3.75
mesoderm transcript	1.29	1.77	2.09	3.12
*tropomyosin 1alpha*	1.36, 1.37, 1.32	2.2, 2.17, 2.1, 1.9	1.42, 1.38, 1.35	3.68, 3.64, 2.94
alpha fetoprotein	3.0	3.09	3.61	3.22
POU domain class 5	0.66	0.00	−2.01	−5.18
*Arc*	−2.37	−1.10	−0.79	−1.39
Ran small G protein	−2.28	−0.23	−0.40	−1.65

The monolayer cultures of ES cells were treated with or without retinoic acid (RA) for 4 days and mRNA was isolated for expression array analysis. A selected list of genes of interest with large-fold expression changes following RA treatment is presented. The fold-changes in expression between with or without RA can be calculated as 2n. The number “n” is listed in the table. Multiple numbers in a category indicate several cDNA entries of the same gene on the cDNA chip. The presence of hybridization signal for a GATA transcript in cells that were homozygous knockout of the GATA gene is likely due to the presence of the mutant GATA transcripts that are inactive. The authors will provide the full data of the expression array experiments if requested. The complete list of differentially expressed genes will be found online: http://www.ncbi.nlm.nih.gov/projects/geo/, Accession “GPL4486”.
